# Supplementary material for: Lactoferrin-Derived Peptides as a Control Strategy against Skinborne Staphylococcal Biofilms
Source: Biomedicines. 2020 Sep 1;8(9):323. doi: 10.3390/biomedicines8090323 (PMC7554924; doi:10.3390/biomedicines8090323)
Supplement: Supplementary file 1 [file biomedicines-08-00323-s001.pdf]

biomedicines

MDPI

Supplementary material

Figure S1. Antimicrobial activity of bovine lactoferrine hydrolysate (HLF) at different concentrations (0, 0.625, 1.25, 2.5, 5, 10 and 20 mg/mL) against Staphylococcus spp. strains forming a biomass of biofilm higher than 0.4 (as $OD_{\lambda = 570\ nm}$) after 24 and 48 h of incubation. The uppercase letters above bars show homogeneous subgroups ($P > 0.05$) based on Kruskal-Wallis analysis followed by Dunn's *post hoc* tests (N = 3).

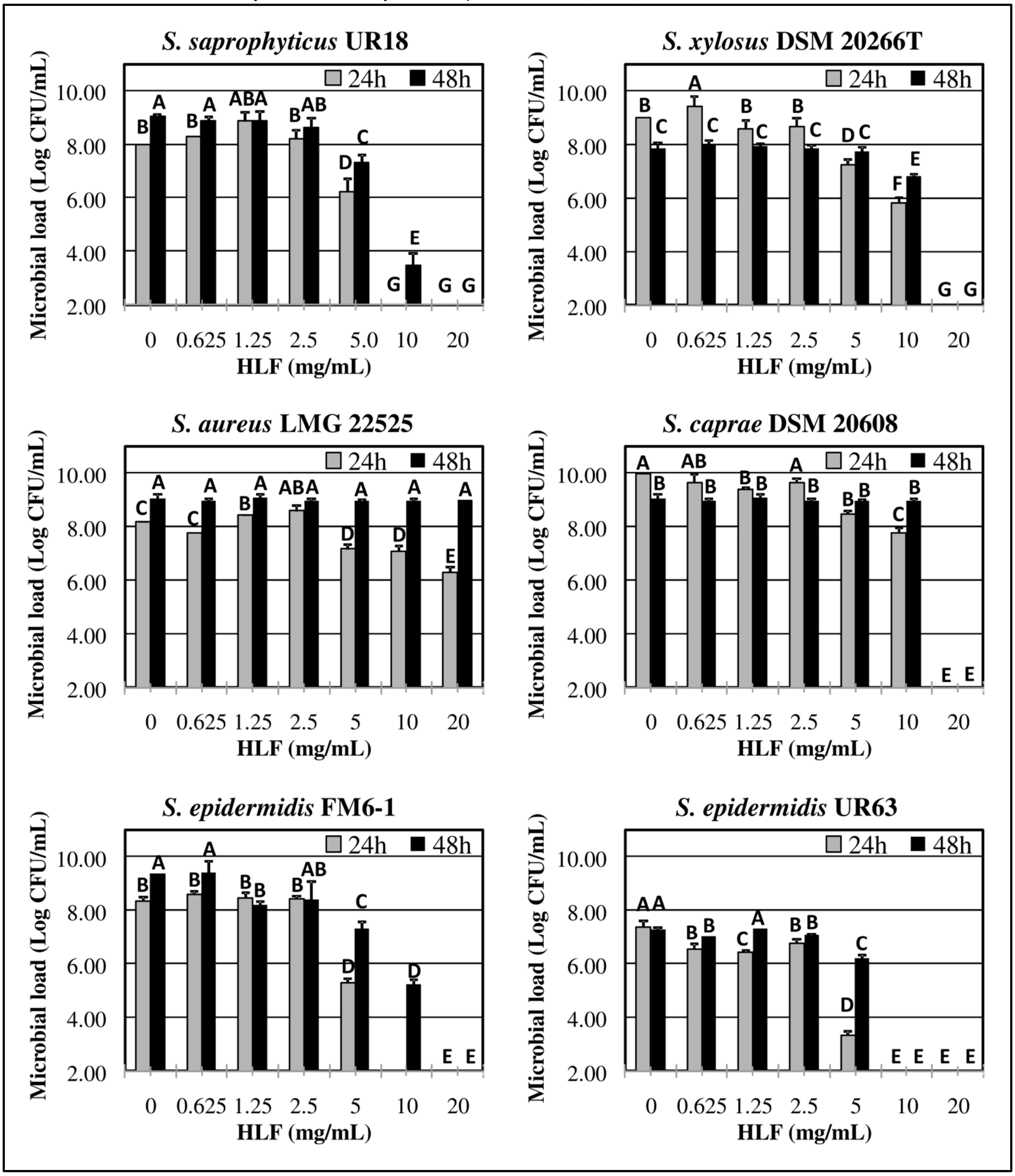

biomedicines

MDPI

Figure S2. Cross-correlation factor (Xcorr) of ions identified by LC-MS/MS in bovine lactoferrin hydrolysate (HLF) and attributed to the bovine lactotransferrin protein *Bos taurus* (Accession number GenBank: AAA30610.1.).

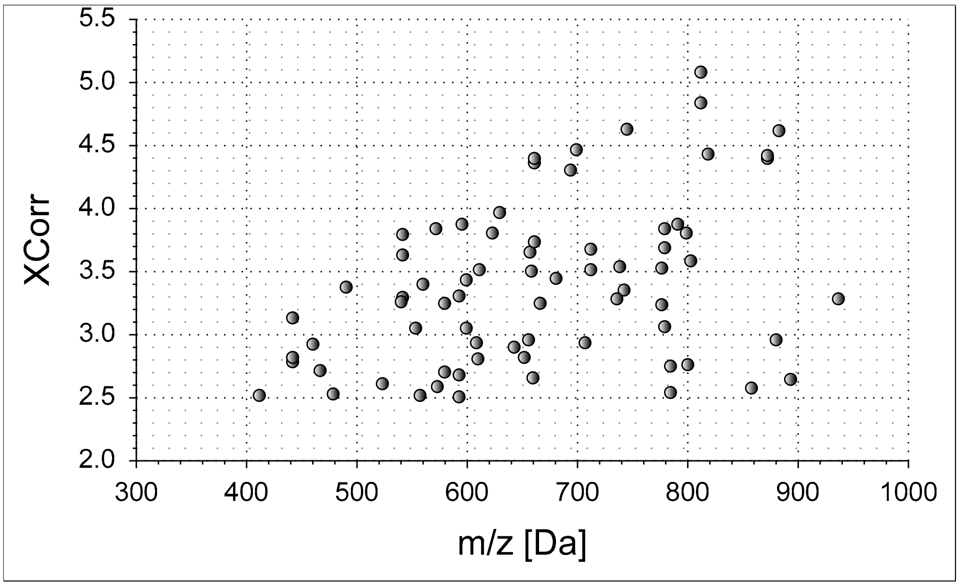

biomedicines

MDPI

Figure S3. A) Gel Filtration Chromatography (GFC) of bovine lactoferrin hydrolysate (HLF). Numbers from 1 to 5 represent the collected fractions tested for their antimicrobial activity. LFmpin: Lattoferrampin; B) Microbial load of *S. epidermidis* UR63 cultures treated with different GFC fractions or water (control) and grown for 0, 4, 8 and 24 days. Bars represent mean values ± standard deviations. Values statistically different ($P < 0.05$ and 0.0001) from control at the respective incubation time were indicated with asterisks * and **, respectively (Student's t–test).

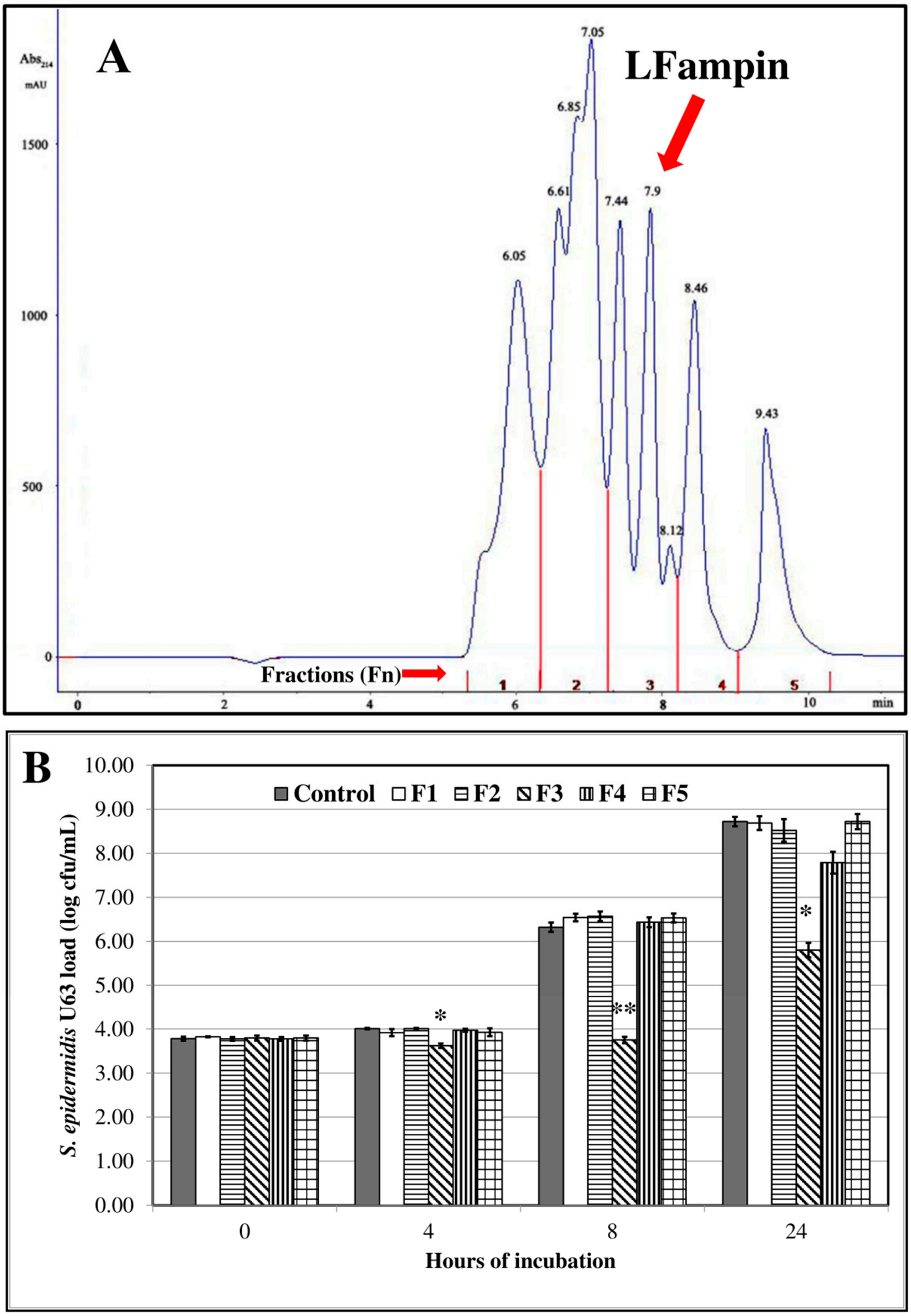

Table S1. Peptides identified from bovine lactoferrin hydrolysate by LC-MS/MS (Proteins: Lactotransferrin *Bos taurus*, Accession N°. P24627; Score 222,02; Coverage 0,347).

Sequence	XCorr	m/z [Da]	MH+ [Da]	Charge	ΔM [ppm]	RT [min]	Missed Cleavages	PSMs	Proteins	Protein Groups	Modifications
ESLEPLQGAVAKF	5,61	463,65222	1388,94211	3	144,02	15,70	0	3	1	1	
LSKAQEKFGKNKSRSFQL	4,84	1048,61572	2096,22417	2	30,04	1,57	0	1	1	1	
ESLEPLQGAVAKFF	4,60	768,27052	1535,53377	2	-180,22	23,30	0	1	1	1	
TESLEPLQGAVAKF	4,50	745,48071	1489,95415	2	110,33	16,00	0	1	1	1	
VLKGEADALNLDGGY	4,49	767,99841	1534,98955	2	139,88	12,36	0	2	1	1	
VLKGEADALNLDGGYIY	4,40	905,86157	1810,71587	2	-113,97	16,82	0	1	1	1	
SWTESLEPLQGAVAKF	4,38	881,77686	1762,54643	2	-201,23	21,11	0	1	1	1	
KKADAVTLDGGM	4,34	603,37872	1205,75017	2	108,35	2,60	0	2	1	1	
EKKADAVTLDGGM	4,21	667,76492	1334,52257	2	-104,58	2,93	0	1	1	1	
IIPMGILRPYLSWT	4,19	830,25256	1659,49785	2	-259,91	25,98	0	2	1	1	
KGEADALNLDGGY	4,17	661,86493	1322,72258	2	75,78	10,38	0	1	1	1	
WIIPMGILRPYLSWT	4,16	923,36255	1845,71782	2	-157,48	31,42	0	1	1	1	
KKADAVTLDGGMV	4,09	652,68075	1304,35422	2	-255,86	5,72	0	1	1	1	
KFGKNKSRSFQL	4,01	720,46851	1439,92974	2	81,89	1,47	0	1	1	1	
VLKGEADALNL	3,97	571,88885	1142,77043	2	112,71	10,24	0	1	1	1	
WIIPMGILRPYL	3,94	736,59991	1472,19255	2	233,05	28,14	0	2	1	1	
FQLFGSPPGQRDLL	3,93	788,02100	1575,03472	2	128,30	19,86	0	2	1	1	
WIIPMGILRPYLS	3,87	779,77116	1558,53505	2	-222,29	27,60	0	1	1	1	
SLEPLQGAVAKF	3,83	630,41064	1259,81401	2	90,91	14,87	0	1	1	1	
IAEKKADAVTLDGGM	3,78	759,88892	1518,77056	2	-8,41	3,57	0	5	1	1	
SFQLFGSPPGQRDLL	3,75	831,27403	1661,54079	2	-194,92	19,90	0	2	1	1	
LRIPSKVDSAL	3,67	599,97430	1198,94133	2	188,39	7,82	0	1	1	1	
IAEKKADAVTLDGGm	3,64	512,17292	1534,50420	3	-178,58	3,97	0	2	1	1	M15(Oxidation)
VLKGEADALNLDGG	3,62	686,34929	1371,69130	2	-14,74	9,12	0	1	1	1	
LFKDSALGF	3,59	499,33691	997,66655	2	131,50	14,73	0	1	1	1	
KKADAVTLDGGMVF	3,58	726,36017	1451,71306	2	-29,83	12,21	0	1	1	1	
FQLFGSPPGQRDL	3,56	731,18512	1461,36297	2	-263,86	16,16	0	1	1	1	

Table S1. To be continued.

Sequence	XCorr	m/z [Da]	MH+ [Da]	Charge	ΔM [ppm]	RT [min]	Missed Cleavages	PSMs	Proteins	Protein Groups	Modifications
LTTLKNLRETAE	3,54	694,92407	1388,84087	2	47,84	2,92	0	2	1	1	
WIIPMGILRPY	3,43	679,93231	1358,85735	2	67,67	24,88	0	1	1	1	
SFQLFGSPPGQRDL	3,39	774,77088	1548,53449	2	-158,92	16,53	0	1	1	1	
SAGWIIPMGILRPYL	3,36	843,94141	1686,87554	2	-38,26	28,41	0	1	1	1	
LTTLKNLRETAEE	3,35	759,27002	1517,53276	2	-187,32	4,75	0	1	1	1	
FGSPPGQRDLL	3,22	593,91730	1186,82732	2	173,37	9,48	0	2	1	1	
TTLKNLRETAE	3,22	638,39136	1275,77544	2	66,69	2,03	0	1	1	1	
KKADAVTLDGGm	3,21	611,17844	1221,34959	2	-216,85	2,62	0	1	1	1	M12(Oxidation)
YLGSRYLTTL	3,21	593,84198	1186,67668	2	25,29	14,08	0	1	1	1	
LSWTESLEPLQG	3,18	680,18228	1359,35728	2	-236,76	20,90	0	1	1	1	
FKSETKNLL	3,16	540,36450	1079,72173	2	103,87	2,46	0	1	1	1	
RPYLSWTESLEPLQG	3,15	888,27721	1775,54714	2	-196,67	18,82	0	1	1	1	
DALNLDGGY	3,15	469,23199	937,45671	2	32,56	14,24	0	1	1	1	
QLFGSPPGQRDLLF	3,11	787,77161	1574,53594	2	-188,43	19,46	0	1	1	1	
FKDSALGF	3,05	442,85147	884,69567	2	276,22	10,54	0	1	1	1	
QLFGSPPGQRDLL	3,00	714,18420	1427,36113	2	-282,40	14,72	0	1	1	1	
DRTAGWNIPMGL	2,97	665,68152	1330,35576	2	-226,67	20,06	0	1	1	1	
FKDSALGFL	2,96	499,35776	997,70824	2	173,27	16,66	0	1	1	1	
WIIPmGILRPYL	2,96	744,51013	1488,01299	2	113,31	24,61	0	2	1	1	M5(Oxidation)
VLKGEADAL	2,96	458,27643	915,54558	2	33,80	3,08	0	1	1	1	
GRSAGWIIPMGIL	2,94	685,76592	1370,52457	2	-172,79	24,07	0	1	1	1	
ILRPYLSWT	2,93	574,75974	1148,51220	2	-116,75	15,86	0	1	1	1	
LKNLRETAE	2,91	537,31287	1073,61846	2	21,85	1,30	0	1	1	1	
ENLPEKADRDQYE	2,90	803,93396	1606,86064	2	78,55	2,03	0	1	1	1	
VLLHQQAL	2,86	461,33380	921,66033	2	117,90	5,35	0	1	1	1	
WIIPmGILRPYLSW	2,86	880,86013	1760,71298	2	-137,87	28,18	0	1	1	1	M5(Oxidation)
AKLGGRPTYEE	2,85	610,83459	1220,66191	2	28,58	1,44	0	1	1	1	
LYLGSRYLTT	2,84	593,91968	1186,83208	2	156,22	11,91	0	1	1	1	
RSVDGKEDL	2,80	509,81433	1018,62139	2	103,05	1,29	0	1	1	1	

Table S1. To be continued.

Sequence	XCorr	m/z [Da]	MH+ [Da]	Charge	ΔM [ppm]	RT [min]	Missed Cleavages	PSMs	Proteins	Protein Groups	Modifications
WAKNLNREDF	2,77	646,92047	1292,83367	2	151,14	5,05	0	1	1	1	
ENLPEKADRDQY	2,76	739,33594	1477,66460	2	-18,42	1,91	0	1	1	1	
WAKNLNRED	2,72	573,31787	1145,62847	2	51,16	1,43	0	1	1	1	
SWTESLEPLQGA	2,71	659,13477	1317,26225	2	-280,83	17,88	0	1	1	1	
LKNLRETAEE	2,68	601,76124	1202,51521	2	-101,78	1,37	0	1	1	1	
RSVDGKEDLIWKL	2,66	779,77116	1558,53505	2	-207,76	11,33	0	1	1	1	
KKADAVTL	2,60	423,27240	845,53752	2	33,57	1,88	0	1	1	1	
IIPMGILRPYL	2,60	643,38251	1285,75774	2	-9,65	22,06	0	1	1	1	
FVKETTVF	2,59	485,82391	970,64055	2	119,61	8,28	0	1	1	1	
VKETTVF	2,59	412,29477	823,58226	2	153,27	4,16	0	2	1	1	
KSETKNLL	2,57	466,77399	932,54070	2	-0,50	2,05	0	2	1	1	
VFEAGRDPYKLRPVAA	2,53	894,77757	1788,54787	2	-239,16	6,92	0	1	1	1	
IAEKKADAVT	2,51	523,17353	1045,33978	2	-238,27	1,18	0	1	1	1	
FGSPPGQRDL	2,46	537,27185	1073,53642	2	-0,99	3,39	0	2	1	1	
EPLQGAVAKF	2,38	530,36182	1059,71636	2	125,48	9,79	0	1	1	1	
YLGSRYLTT	2,34	537,38513	1073,76299	2	186,62	8,42	0	1	1	1	
RPYLSWTESLEPLQGAV	2,32	973,28195	1945,55662	2	-228,86	21,13	0	1	1	1	
PPGQRDLL	2,31	448,31577	895,62425	2	139,14	9,50	0	1	1	1	
ENLPEKADRDQYEL	2,17	860,19232	1719,37737	2	-256,56	6,17	0	1	1	1	
KGEADALNLDGGYIY	2,11	799,77228	1598,53728	2	-145,42	15,75	0	1	1	1	
